# Effects of a robot-assisted training of grasp and pronation/supination in chronic stroke: a pilot study

**DOI:** 10.1186/1743-0003-8-63

**Published:** 2011-11-16

**Authors:** Olivier Lambercy, Ludovic Dovat, Hong Yun, Seng Kwee Wee, Christopher WK Kuah, Karen SG Chua, Roger Gassert, Theodore E Milner, Chee Leong Teo, Etienne Burdet

**Affiliations:** 1 Department of Mechanical Engineering, National University of Singapore, Singapore, Singapore; 2 Rehabilitation Engineering Lab, ETH Zurich, Zurich, Switzerland; 3 Department of Rehabilitation Medicine, Tan Tock Seng Hospital, Singapore, Singapore; 4 Department of Kinesiology and Physical Education, McGill University, Montreal, Canada; 5 Department of Bioengineering, Imperial College of Science, Technology and Medicine, London, UK

## Abstract

**Background:**

Rehabilitation of hand function is challenging, and only few studies have investigated robot-assisted rehabilitation focusing on distal joints of the upper limb. This paper investigates the feasibility of using the *HapticKnob*, a table-top end-effector device, for robot-assisted rehabilitation of grasping and forearm pronation/supination, two important functions for activities of daily living involving the hand, and which are often impaired in chronic stroke patients. It evaluates the effectiveness of this device for improving hand function and the transfer of improvement to arm function.

**Methods:**

A single group of fifteen chronic stroke patients with impaired arm and hand functions (Fugl-Meyer motor assessment scale (FM) 10-45/66) participated in a 6-week 3-hours/week rehabilitation program with the *HapticKnob*. Outcome measures consisted primarily of the FM and Motricity Index (MI) and their respective subsections related to distal and proximal arm function, and were assessed at the beginning, end of treatment and in a 6-weeks follow-up.

**Results:**

Thirteen subjects successfully completed robot-assisted therapy, with significantly improved hand and arm motor functions, demonstrated by an average 3.00 points increase on the FM and 4.55 on the MI at the completion of the therapy (4.85 FM and 6.84 MI six weeks post-therapy). Improvements were observed both in distal and proximal components of the clinical scales at the completion of the study (2.00 FM wrist/hand, 2.55 FM shoulder/elbow, 2.23 MI hand and 4.23 MI shoulder/elbow). In addition, improvements in hand function were observed, as measured by the Motor Assessment Scale, grip force, and a decrease in arm muscle spasticity. These results were confirmed by motion data collected by the robot.

**Conclusions:**

The results of this study show the feasibility of this robot-assisted therapy with patients presenting a large range of impairment levels. A significant homogeneous improvement in both hand and arm function was observed, which was maintained 6 weeks after end of the therapy.

## Background

Stroke is one of the leading causes of adult disability. While there is strong evidence that physiotherapy promotes recovery, conventional therapy remains suboptimal due to limited financial and human resources, and there are many open questions, e.g. when therapy should be started, how to optimally engage the patient, what is the best dosage, etc. [1-3]. Furthermore, exercise therapy of the upper limb has been shown to be only of limited impact on arm function in stroke patients [4].

Robot-assisted rehabilitation can address these shortcomings and complement traditional rehabilitation strategies. Robots designed to accurately control interaction forces and progressively adapt assistance/resistance to the patients' abilities can record the patient's motion and interaction forces to objectively and precisely quantify motor performance, monitor progress, and automatically adapt therapy to the patient's state.

Studies with robots such as the MIT-Manus, the ARM Guide or the MIME have demonstrated improved proximal arm function after stroke [5-8], although these improvements did not transfer to the distal arm function which is necessary for most Activities of Daily Living (ADL) [9-11]. Robot-assisted training which specifically targets the hand might be required to achieve significant improvements in hand function. Furthermore, several studies indicate a generalization effect of distal arm training, e.g. hand and wrist, on proximal arm function, i.e. elbow and shoulder, which may lead to improved control of the entire arm [10,12,13].

We therefore focused on robot-assisted rehabilitation of the hand, adopting a functional approach based on the combined training of grasping and forearm pronation/supination, two critical functions for manipulation. This paper presents the results of a pilot study using the *HapticKnob*, a portable end-effector based robotic device to train hand opening/closing and forearm rotation. In contrast to robotic devices based on exoskeletons attached to the arm [14], the *HapticKnob *applies minimal constraints to the different joints of the upper arm, thus corresponding to situations encountered during ADL. The forearm rests on an adjustable padded support, while the shoulder and upper arm are not restrained.

The objectives of this pilot study were to determine the feasibility of training chronic stroke patients with the *HapticKnob*, and to reduce motor impairment of the upper limb in a safe and acceptable manner. Although a few studies have investigated post-stroke rehabilitation of the hand [12,13], ours is the first to use robot-assisted training that combines grasp and forearm pronation/supination to perform functional tasks. With this pilot study, we tested the hypothesis that training the hand using this functional approach improves function of the entire arm.

## Methods

### Subjects

Fifteen subjects (55.5 ± 14.6 years, 7 men) with chronic post-stroke hemiparesis, who were at least 9 months post-stroke (mean 597.5 ± 294.1 days) were recruited for this study (Table 1). The sample size was limited by the number of patients that could be enrolled over the duration of the project. Ethical approval was obtained from Tan Tock Seng Hospital (TTSH) Institutional Review Board before subjects were approached for screening and informed consent (DSRB A/07/715). Subjects presented slight to severe residual arm impairment and had completed the initial stroke rehabilitation program at TTSH. Inclusion criteria were subjects aged between 21 and 85 years with impaired hand opening but capable of partial hand and arm movement corresponding to proximal upper limb motor power (shoulder-elbow) graded 3-5 out of 5 on the Oxford Medical Research Council (MRC) scale, distal upper extremity motor power (wrist-hand) graded 0-3 out of 5 on the MRC scale, and initial Fugl-Meyer motor assessment scale (FM) for the upper extremity graded between 10-45 points out of 66. Furthermore, subjects should have the ability to understand the instructions and to perform exercises with the *HapticKnob*, and to give own consent. Exclusion criteria were medical or functional contraindications to intensive training, upper limb pain > 4/10 on a Visual Analogue Scale (VAS), upper limb spasticity > 2 on the Modified Ashworth Scale (MAS), spastic dystonia or contractures, poor skin condition over hand and wrist, and visual spatial neglect based on clinical judgment.

**Table 1:** Demographic characteristics and stroke subtypes (N = 15)

subject	age (years)	gender (M/F)	time post- stroke (days)	initial Fugl-Meyer score	stroke type	lesion site
A1	55	M	929	32	hemorrhagic	left basal ganglia
A2	68	M	1064	34	hemorrhagic	right basal ganglia
A3	48	M	323	13	hemorrhagic	left basal ganglia
A4	46	M	679	34	ischemic	right temporal, basal ganglia, corona radiata, thalamus
A5	61	F	458	43	ischemic	right frontal-temporal, insula
A6	78	F	831	16	ischemic	right temporal, basal ganglia
A7	63	F	934	42	ischemic	right basal ganglia
A8	73	F	319	27	hemorrhagic	left basal ganglia
A9	43	F	417	37	hemorrhagic*	right frontal lobe
A10	71	F	318	40	ischemic	right corona radiata, basal ganglia
A11	65	F	271	33	ischemic*	right basal ganglia, corona radiata, external capsule frontal-parietal, thalamus
A12	31	M	297	35	hemorrhagic	right parietal lobe
A13	55	M	480	14	hemorrhagic	right basal ganglia
A14	44	F	627	41	hemorrhagic	left basal ganglia
A15	32	M	1041	45	hemorrhagic	right basal ganglia
mean	55.5 ± 14.6	-	597.5 ± 294.1	32.4 ± 10.5	-	-

* recurrent stroke

all subjects were right handed

### The HapticKnob

**Figure 1:**
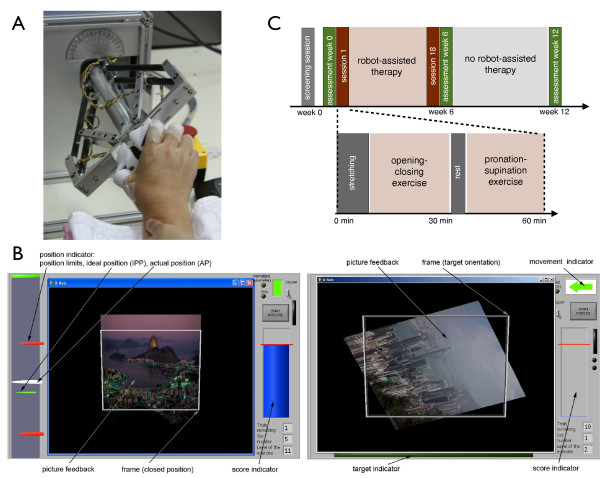
**The HapticKnob robot and the proposed therapy protocol**. A: Stroke subject training on the HapticKnob. B: Visual feedback of the opening/closing (left) and pronation/supination (right) exercises, where subjects have to squeeze, respectively orient the picture into a white frame by grasping, respectively turning the *HapticKnob*. C: Details of therapy and session protocol.

The *HapticKnob *[15] is a two degrees-of-freedom (DOF) robotic device used to train grasping in coordination with pronation/supination of the forearm. These functions are crucial for object manipulation during ADL, e.g. turning a doorknob, pouring water into a glass, etc., and are among the distal arm functions stroke subjects miss the most. The design of the *HapticKnob *is based on an end-effector approach, where the robot interacts with the user at the level of the hand (Figure 1A). It can generate assistive or resistive forces of up to 50*N *in both hand opening and closing and torques of up to 1.5*Nm *in pronation and supination. While these values are far from the maximum force/torque a healthy subject can generate (about 450*N *in grasping and 20*Nm *in pronation/supination), they are sufficient to provide challenging exercises for stroke patients and simulate typical ADL manipulation tasks [15]. Force sensors (MilliNewton 2N, Thick Film Technology group, EPFL, Switzerland) are incorporated under each finger support to measure grasping forces of up to 30*N *applied on the knob. Fixtures of different size and shape can be attached to the *HapticKnob *to train different hand functions such as power grasp, pinch or lateral pinch. In the study presented in this paper, a disk with a diameter of 6*cm *was mounted at the end effector of the robot. During interaction with the robot, various force effects can be implemented, e.g. to resist or assist the movement, and the range of motion and force/torque amplitude can be modified to automatically adapt the training parameters to the user's level of impairment. An adaptable, padded arm support is fixed in front of the robot. The *HapticKnob *is controlled using a PC running LabView 8.2 (National Instruments, USA).

Two simple task-oriented exercises corresponding to typical ADL were implemented on the *HapticKnob*. One first objective is to reduce hand impairment, i.e. spasticity and limited active finger range-of-motion (ROM), by providing passive assistance similar to stretching [13] for hand opening movements that often are too difficult for perform. Active force production is promoted to increase muscle strength, improve control of the impaired limb and facilitate acquisition and retention of skills.

(i) *opening/closing exercise*, training extension then flexion of the fingers to simulate grasping of an object. In a first phase of the exercise, the robot opened the fingers to an extended position adapted to the subject's range of motion (ROM), which was selected between 10 and 15 *cm *from the tip of the thumb to the tip of the opposing fingers for the subjects of this study. At the end of the opening phase, the robot maintained the position for three seconds during which subjects were asked to relax and apply minimal grasping force. An audio signal indicated the beginning of the closing phase, which required the subject to actively flex the fingers against a resistive load between 0 to 30*N *generated by the robot, according to the difficulty level of the exercise. To train grasping force control, subjects were asked to smoothly close the hand by following a reference position profile (RPP) displayed on the monitor (Figure 1B), which corresponded to a fifth order polynomial defining a minimal jerk movement between the open and closed positions, as natural movements tend to follow [16].

(ii) *pronation/supination exercise*, training forearm rotation and coordination between grasping and turning required to manipulate knobs [15]. In this exercise, subjects were asked to supinate or pronate the forearm towards a specific target orientation, while the linear DOF of the *HapticKnob *remained in the closed position. This task required the subjects to produce accurate rotation movements, reach a [-1°, 1°] position window around the target in a minimal time, and remain there for 2 seconds (without exiting). This window was adapted to the human discrimination threshold in orientation, which is between 0.4-1° [17]. In this study, the amplitude of forearm rotation was selected between 25° and 45°, corresponding to the subjects' ROM. In addition, a resistive torque load adapted to the subject's impairment level and comprised between 0 and 1*Nm *was applied by the robot during the exercise in order to require the subject to hold the knob firmly during the movement.

During training, interactive and intuitive visual feedback was provided to the subject to promote concentration and motivation. A picture that was stretched in the open/close exercise and rotated in the pronation/supination exercise (Figure 1B), in function of the movement performed with the subject was displayed on the monitor, while the target position to reach was represented by a white frame. In addition, exercises were presented as games with a score calculated based on the timing and precision of the task. This score was provided as feedback to the subject, and used to adjust the level of difficulty of the task [18]. During each trial, position and force signals were sampled at a frequency of 100*Hz *and stored for post-processing.

### Training protocol

Robot-assisted therapy consisted of 18 one-hour sessions of training with the *HapticKnob *over a period of 6 weeks. Prior to the first therapy session, a preliminary test session was performed to ensure that subjects were able to interact with the robot and understood the exercises. All sessions were supervised by an occupational therapist. Before starting the exercises, 10 minutes were devoted to stretching to reduce muscle tone and to comfortably position the subject. Each exercise consisted of 5 sets of 10 trials, lasting about 25 minutes. There was a short rest period between each set to prevent muscle fatigue and a 5-minute break between the two exercises to stretch and relax arm muscles (Figure 1C).

During therapy sessions, subjects sat in an upright position, placed the forearm on the padded support and grasped the *HapticKnob *with the hand. The arm support and the height of the table on which the robot was placed were adjusted to offer the subject a comfortable position, with the arm resting on the support during the experiment, the shoulder abducted at 40° and the elbow flexed at 90°. No support was provided at the level of the proximal arm, so that subjects could position and move their upper arm freely. Possible compensatory trunk movement or abnormal wrist hyper-flexion were monitored and manually prevented by the occupational therapist supervising the therapy. If the subject had difficulty holding the knob, Velcro^® ^bands were used to prevent fingers and thumb from slipping off the knob.

### Robotic outcome measures

Kinematic data collected by the *HapticKnob *can be used to evaluate motor performance in the functional tasks trained with the device. To evaluate hand motor control during the opening/closing exercise, the mean absolute error ε_p _between the RPP and the position waveform during closing. Motion smoothness was estimated from the number of zero crossings of the acceleration n_0 _(indicating putative velocity submotions [19]), normalized by the duration of the closing movement. In the pronation/supination exercise, coordination between grasping and fine forearm orientation was assessed by the time t_r _required to reach the target window, and the time t_a _to adjust the angular position once the target is reached for the first time [20]. Robotic data were processed using Matlab R2010a (The MathWorks, Inc.).

### Clinical outcome measures

Subjects were assessed at three times during the study: prior to the beginning of the therapy (week 0), at its completion (week 6), and 6 weeks post-therapy (week 12). Between week 6 and week 12, patients did not receive any further rehabilitation therapy focusing on upper extremity motor function. All assessments were done by an occupational therapist not involved in the *HapticKnob *training. The primary objective of the proposed training being to decrease impairment and upper limb improve motor function, the Fugl-Meyer motor assessment for the upper extremity (FM, range (0-66) [21]) and the Motricity Index (MI) for motor function of the upper limb were selected as primary outcome measures. FM scores were subdivided into wrist-hand scores (0-24), and shoulder-elbow scores including coordination (0-42). MI scores were converted from raw scores to subscores with a total of 100 points [22]. Similarly to FM scores, MI scores were subdivided into hand scores (0-33) and shoulder-elbow scores (0-66).

Secondary outcomes were selected to investigate independent neurophysiological changes not covered by the primary outcome measures, and included the Motor Assessment Scale (MS, range (0-18)) to assess everyday motor function involving the arm and hand [23], the Modified Ashworth Scale as a measure of spasticity in shoulder abductors, elbow, wrist, finger and thumb flexors (modified MAS, range (0-5) [24]), the Functional Test of Hemiparetic Upper Extremity (FTHUE, range (0-7) [25]), the Nine Hole Peg Test (NHPT) [26], and grip force measurement using a Jamar Grip Dynamometer. Pain was assessed using a Visual Analog Scale (VAS range (0-10)) and the subject provided a score of satisfaction with the therapy (1 = 'poor', 2 = 'satisfactory', 3 = 'good' or 4 = 'excellent').

### Data analysis

Data were analyzed using SPSS v18 statistical analysis package (IBM). Due to the small sample size, non-parametric tests were used to investigate differences in means. Statistical difference was first investigated for each clinical measure using a two-tailed Friedman test. Bonferroni correction was used to compensate for the two primary outcome measures of upper limb motor function, so that all tests were applied using a 0.025 significance level. Post-hoc analysis for possible differences between baseline discharge and follow-up was then performed using Wilcoxon signed rank tests (0.05 significance level). For the secondary outcome measures, no Bonferroni correction was used to correct for the multiple assessments, as these are assumed to be independent. For the robotic measures, Wilcoxon tests with a 0.05 significance level were used to investigate differences in means between results of the first and last training sessions.

## Results

All of the 15 post-stroke subjects completed the pilot study, consisting of 18 hours of *HapticKnob *training over 6 weeks. However, subject A12 had to stop therapy for a week due to an unrelated fall at home. Further, A11 had severe concentration problems and suffered from depression Therefore, data from these two subjects were excluded from the analysis.

Results of primary outcome measures are presented in Table 2. There were significant increases in FM (Friedman p < 0.001) and MI (Friedman p < 0.001) scores, indicating improved upper limb motor function and strength. There were improvements in proximal and distal subsections of the two primary outcome measures. These improvements were significant in the distal subportion of the FM (Friedman p < 0.002) and in the proximal subsection of the MI (Friedman p < 0.002).

**Table 2:** Primary outcome measures at week 0, week 6 and week 12 for the 13 subjects that were retained for data analysis (mean ± std), and p-values of the statistical analysis (Friedman tests with 0.025 significance level and post-hoc Wilcoxon tests with 0.05 significance level).

Primary outcome measures	week 0	week 6	week 12	Friedman test	Wilcoxon test 0-6	Wilcoxon test 0-12
Fugl-Meyer total (normal = 66)	32.15 ± 11.31	35.15 ± 12.05	37.00 ± 11.21	0.001	0.009	0.005
Subportion wrist/hand (normal = 24)	8.23 ± 4.38	9.15 ± 4.74	10.23 ± 5.40	0.002	0.018	0.009
Subportion shoulder/elbow (normal = 42)	23.92 ± 8.54	26.00 ± 8.81	26.77 ± 7.56	0.042	n.a.	n.a.

Motricity Index (normal = 100)	50.62 ± 15.41	55.16 ± 17.99	57.46 ± 16.26	0.001	0.025	0.003
Subportion hand/fingers (normal = 33)	12.46 ± 16.25	13.54 ± 10.84	14.69 ± 11.33	0.551	n.a.	n.a.
Subportion shoulder/elbow (normal = 66)	37.15 ± 9.94	40.62 ± 11.74	41.38 ± 11.16	0.002	0.027	0.011

**Figure 2:**
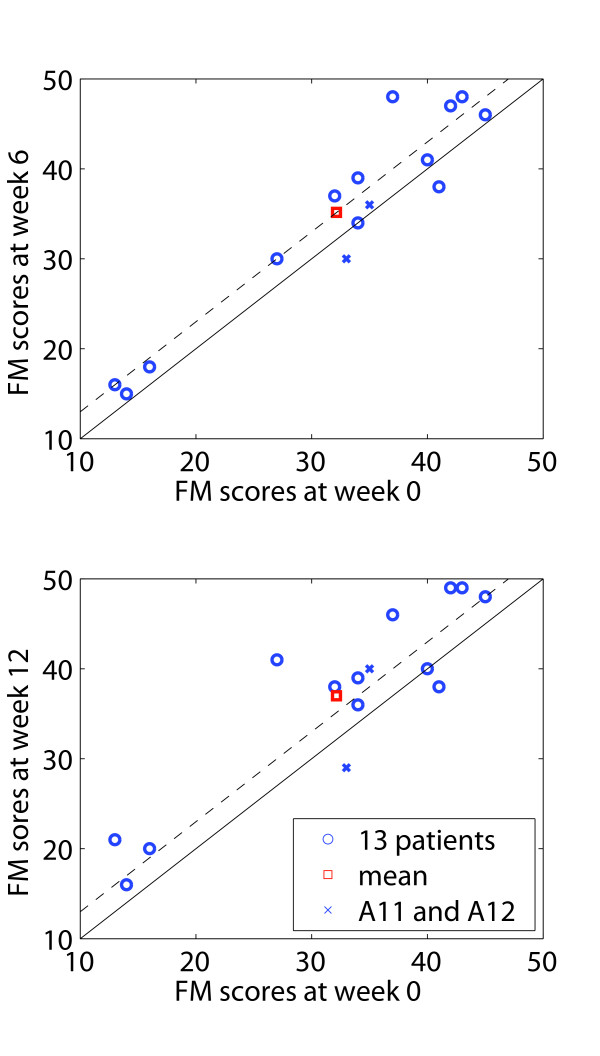
**Results of the Fugl-Meyer scores for the upper extremity**. Comparison of Fugl-Meyer (FM) scores for the upper extremity between week0/week6, and week0/week12. Circles represent results of the 13 participants included in the data analysis, squares represent the mean over the 13 subjects, and crosses represent results of subjects A11 and A12, who had a break in the treatment and were thus excluded from the analysis. Dashed lines illustrate a 3-point improvement on the FM considered as a functionally meaningful improvement [10].

At the end of the robot-assisted therapy (week 6) subjects had improved 3.00 points (+9.3%) on average on the FM scale (p < 0.009) with a maximum improvement of 11 points for subject A9 (Figure 2). There were improvements in both subportions of the FM score, with an average increase of 0.92 points (+11.2%, p < 0.018) for the wrist-hand subsection of the FM. Similarly, subjects improved 4.54 points (+9.0%, p < 0.025) on the MI. There was no significant effect of the age group or gender.

**Figure 3:**
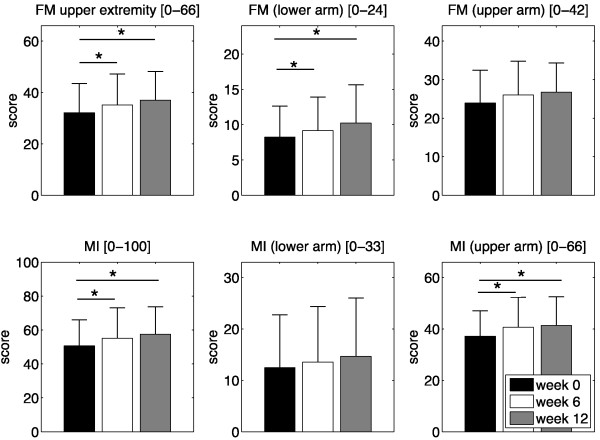
**Primary outcome measures**. Evolution of Fugl-Meyer (FM) scores for the upper extremity and Motricity Index (MI) scores for the 13 subjects that were retained for data analysis (mean ± std), with details of sections related to the lower and upper arm (*p < 0.05).

Six weeks after completion of the robot-assisted therapy (week 12) the average gain in FM was 4.85 points (+15.1%, p < 0.005). The distal arm showed greater percentage improvement than the proximal arm during the follow-up period with an average increase of 2.00 points (+24.3%, p < 0.009) compared to 2.85 points (+11.9%). The results were similar for MI scores, with an average increase of 6.85 points (+13.5%, p < 0.003). Although not statistically significant, distal components of the MI (i.e. hand-fingers) improved on average by 2.23 points (+17.9%) while proximal components (i.e. shoulder-elbow) improved by 4.23 points (+11.4%, p < 0.011). Figure 3 illustrates the evolution of primary outcome parameters after the 6 weeks of robot-assisted therapy and the 6-week follow-up.

Table 3 summarizes results for the secondary outcome measures. There was significant increase in the MS (Friedman p < 0.004), indicating a slight improvement in functional activities involving the arm and hand. There was an average increase of 1.00 point (+24.5%, p < 0.010) on the MS scale at the completion of the study. Total (summed) upper limb spasticity showed an average reduction of 0.92 on the MAS scale (-11.1%, p > 0.117) at week 6. The reduction was 1.23 points at week 12 which was statistically significant (-14.8%, p < 0.019).

**Table 3:** Secondary outcome measures at week 0, week 6 and week 12 for the 13 subjects that were retained for data analysis (mean ± std), and p-values of the statistical analysis (Friedman tests with 0.05 significance level and post-hoc Wilcoxon tests with 0.05 significance level).

Secondary outcome measures	week 0	week 6	week 12	Friedman test	Wilcoxon test 0-6	Wilcoxon test 0-12
Motor Assessment Scale (normal = 18)	4.08 ± 3.30	5.08 ± 3.30	5.23 ± 3.63	0.004	0.010	0.006
Modified Ashworth Scale (normal = 0)	8.31 ± 3.12	7.38 ± 2.43	7.08 ± 3.15	0.038	0.118	0.019
Functional Test of Hemiparetic Upper Extremity (normal = 7)	2.92 ± 0.64	3.08 ± 0.64	3.08 ± 0.64	0.497	n.a.	n.a.
Nine Hole Peg Test *	n.a.	n.a.	n.a.	n.a.	n.a.	n.a.
Grip force (impaired hand/unimpaired hand) [%] †	21.92 ± 15.26	24.60 ± 12.94	22.02 ± 9.11	0.307	n.a.	n.a.
Pain (Visual Analog Scale)	0.46 ± 1.39	0.00 ± 0.00	0.00 ± 0.00	0.135	n.a.	n.a.
Satisfaction grade		3.08 ± 0.76				

* 12 subjects were unable to perform the NHPT

† 2 subjects were unable to perform grip force measurements with their impaired hand

In addition, there was a 12.3% gain in grip strength ratio (grip strength of impaired hand over unimpaired hand) at week 6, though this change was not significant. There was no significant gain in upper arm function as measured by the FTHUE, which could be explained by the low sensitivity of this categorical scale, and the fact that the tasks comprising the FTHUE required a higher level of hand function than that reached by most subjects. Similarly, only one patient was able to perform the NHPT, compromising the use of this assessment in the present study.

Minimal pain experienced by two subjects at the beginning of the study progressively disappeared during the robot-assisted therapy. Therapy with the *HapticKnob *was well accepted by stroke patients, and 10 out of 13 (76.9%) subjects rated their satisfaction post-training as good or excellent.

**Figure 4:**
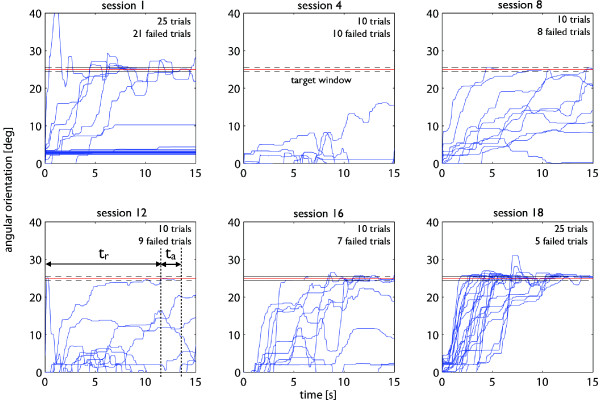
**Example of robotic data collected by the HapticKnob**. Evolution of pronation movements for patient A3 over the course of the therapy at sessions 1, 4, 8, 12, 16 and 18, each line representing one trial. The target angular position window is represented by horizontal dashed lines. Details of parameters extracted from the kinematic data are given in the lower left plot for one successful trial; t_r _is the time required to reach the target window for the first time, and t_a _is the time required to finely adjust the forearm position.

Figure 4 presents representative trials of the pronation/supination exercise performed with the *HapticKnob *for subject A3 over the course of the therapy. A clear increase in the number of successful trials can be seen; movements become faster and more precise, the subject reaches the target pronation angle (25°) at each trial during the last session, while almost no movement was possible in the first session. At the group level, a clear improvement can be observed, with a significant decrease in all indicators (Table 4). Subjects improved control of grasping movement as indicated by a 49.8% decrease in ε_p_, and a 5.1% decrease in n_0 _indicative of smoother movements during the opening/closing exercise. Subjects improved their ability to coordinate hand and forearm function in order to perform the pronation/supination exercise, with a 36.6% decrease in the time t_r _to reach the forearm angle, and a 29.6% decrease in the time to finely tune the position t_a_. This parameter has been shown to be a suitable indicator of upper limb motor function [20].

**Table 4:** Robotic outcome measures at week 0 and week 6 for the 13 subjects that were retained for data analysis (mean ± std), and p-values of the statistical analysis (Wilcoxon tests with 0.05 significance level).

Robotic measures	week 0 (session 1)	week 6 (session 18)	Wilcoxon test
ε_p _[mm]	3.03 ± 2.45	1.52 ± 1.21	0.001
n_0 _[1/s]	6.06 ± 0.52	5.75 ± 0.47	0.033
t_r _[s]	7.40 ± 3.63	4.67 ± 3.24	0.001
t_a _[s]	7.44 ± 3.23	5.24 ± 3.32	0.001

## Discussion

Fifteen chronic stroke subjects with slight to severe arm and hand impairment (mean admission FM of 32.15) performed a robot-assisted rehabilitation therapy program with the *HapticKnob *involving hand opening/closing and forearm pronation/supination. Upper limb motor impairment decreased during the treatment period, as revealed by significant increases in the FM and MI scores, indicating a noticeable improvement of arm and hand function, together with increased upper extremity strength. In the literature, a 3-point improvement on the FM scale is often considered as a minimum impairment change necessary to achieve significant functional gains [10]. Results of the clinical assessments, which were also confirmed by analysis of the robot motion data [18], suggest that intensive use of the forearm and hand in a repetitive robot-assisted training program can improve motor function in chronic stroke subjects even long after completion of conventional therapy (mean 597.5 days post-stroke). Improvement in the robotic parameters suggests that patients could learn to perform the tasks and progressively improve their performance, indicating better hand control and coordination between hand and forearm during the functional tasks proposed during training with the *HapticKnob*. Nevertheless, it is not possible verify whether the improvements observed in the motion data during the training translate to significant gains in functional activities in daily life.

Improvements in arm and hand function were maintained 6 weeks after the completion of the therapy, suggesting a stable improvement of the motor condition. In fact, the primary outcome measures increased further during the 6 weeks after the therapy. The reduction in arm and hand spasticity (although not statistically significant when individual arm components were analyzed) could have facilitated increased use of the impaired hand to perform daily tasks, as could the reduction in pain levels in the two subjects who initially presented with minimal pain. Robot-assisted training may have helped pass a threshold of spontaneous arm use where ADL tasks involving arm and hand are performed at home, thus leading to additional improvement in upper limb motor function and decreasing learned non-use of the affected limb [27]. Subjects reported improvement in ADL at home at the end of the therapy. However, improvements in ADL tasks were not confirmed by corresponding clinical outcome measures, which is also observed in most robot-assisted studies [28]. Changes in fine hand function could not be captured by the NHPT as most patients were unable to complete this dexterity test. A different test such as the Box and Block test [29] should be considered as outcome measure of hand function in future studies.

All 15 chronic stroke subjects were capable of training with the proposed protocol in a safe manner, without experiencing any complication related to the use of the robot, and with significant improvement of motor function in their hand and arm. These results demonstrate the feasibility of using the *HapticKnob *as a rehabilitation tool for chronic stroke patients with a large range of sensorimotor deficits. These results are consistent with results obtained in other robot-assisted studies on upper limb rehabilitation of chronic stroke patients, where improvements of 3.0 to 7.6 points in the FM were found [7,10,11,13,14,30]. However, there is a lack of comparison groups for hand rehabilitation, and the variation in improvement between these studies can be attributed to the differences in experimental protocols, such as intensity and duration of therapy, as well as to initial motor impairment of the stroke subjects involved in the study. In contrast to the devices used in previous studies, the *HapticKnob *is a compact system that could easily be transported and placed in hospitals and homes. It requires only minimal function to place the hand on the robot and thereby makes it accessible to a wide range of subjects, right or left handed, and with various levels of physical impairment, e.g., an initial FM score lower than 15, as demonstrated by this study.

It is likely that the severity of motor impairment is a key factor in rehabilitation outcomes and in the choice of a rehabilitation protocol. Severely impaired subjects may require longer or more intensive therapy to first strengthen the muscles, decrease spasticity and reduce other impairments that limit their performance so as to focus on the restoration of neuromotor pathways without introducing additional complex tasks [31]. In our study, a larger increase in functional assessment scores during therapy was observed in subjects initially with moderate impairment (FM > 35), suggesting that subjects already having some motor function of the arm and hand benefit more from the functional hand therapy with the *HapticKnob*. Nevertheless, this difference between moderately and more severely impaired patients was not statistically significant.

In previous studies, improvement in elbow and shoulder function after training involving these proximal segments did not seem to transfer to the wrist or hand [9-11]. In contrast, the results obtained with the *HapticKnob *indicate that training involving only distal segments of the arm could lead to improvements in both the proximal and distal subsections of the primary outcome measures. Improvement was significant in the hand/wrist subsection of the FM, but not in the shoulder/elbow section after Bonferroni correction. On the other hand, a significant increase was observed in the shoulder/elbow component of the MI, but not in the hand component of MI. Explanations for these seemingly conflicting results include the fact that the distal component of the MI assesses thumb/finger function rather than wrist/hand function and the limited number of subjects included in the study. Nevertheless, a clear positive trend can be observed in all subsections of the two scales, which is confirmed by the secondary outcome measures, with improvement in both arm and hand functional tasks as measured by the MS, and reduced spasticity in all of the arm segments, with the greatest reduction for shoulder abductors and elbow flexors.

These findings support the hypothesis that exercising distal joints of the arm may benefit the proximal joints [10,13,32,33]. As the arm was not fixed but only supported, this effect may be due to a recruitment of all arm segments in a task-oriented way to promote restoration of motor function of the entire arm. In fact, the pronation/supination exercise trains coordination between fingers, wrist and forearm, as subjects are required to firmly grasp the handle and then rotate it and also requires stabilization of the upper arm. Also, distal training requires activation of nerves and muscles that control each segment of the upper limb, and will thus result in proximal as well as distal muscle activity. This is partly because some muscles like the biceps are multi-functional, e.g. supinating the forearm and flexing the elbow and shoulder whereas others are needed to stabilize the more proximal joints even when the forearm is supported. Alternatively, patients may have developed compensatory strategies to achieve forearm pronation/supination with their shoulder, which could account for part of the increase of MI and FM scores. This effect may be monitored in future studies. Finally, these results should be interpreted with caution, as no control group receiving dose-matched conventional or robotic training focusing on the proximal arm segment was included in the study design. Further limitations of the current study include single baseline measure, and absence of a long-term follow-up, which will be considered in future clinical studies.

## Conclusions

The results of this pilot study suggest that upper limb robot-assisted rehabilitation, which currently focuses primarily on training elbow and shoulder movement, would advantageously include training of the hand and fingers, which can be provided using compact desktop robots such as the *HapticKnob*. Whole-arm training, which is a commonly used approach in robot-assisted neurorehabilitation, may not be required, as distal training in a functional way could benefit the whole arm.

## Competing interests

The authors declare that they have no competing interests.

## Authors' contributions

RG contributed to the design and development of the *HapticKnob *and of the experimental protocol. TM, TCL and EB also contributed to the data analysis, and preparation of the manuscript. LD further participated to the clinical evaluation of the *HapticKnob*, while OL contributed to all of these aspects, supervised the robot-assisted therapy at Tan Tock Seng Hospital (TTSH) Rehabilitation center, and prepared the manuscript. HY, SKW, CWKK and KSGC recruited and assessed participants to the clinical study and co-supervised the robot-assisted therapy at TTSH. All authors read and approved the final manuscript.
